# Inhibition of Apoptosis Blocks Human Motor Neuron Cell Death in a Stem Cell Model of Spinal Muscular Atrophy

**DOI:** 10.1371/journal.pone.0039113

**Published:** 2012-06-19

**Authors:** Dhruv Sareen, Allison D. Ebert, Brittany M. Heins, Jered V. McGivern, Loren Ornelas, Clive N. Svendsen

**Affiliations:** 1 Regenerative Medicine Institute, Cedars-Sinai Medical Center, Los Angeles, California, United States of America; 2 Department of Cell Biology, Neurobiology, and Anatomy, Medical College of Wisconsin, Milwaukee, Wisconsin, United States of America; University of Edinburgh, United Kingdom

## Abstract

Spinal muscular atrophy (SMA) is a genetic disorder caused by a deletion of the survival motor neuron 1 gene leading to motor neuron loss, muscle atrophy, paralysis, and death. We show here that induced pluripotent stem cell (iPSC) lines generated from two Type I SMA subjects–one produced with lentiviral constructs and the second using a virus-free plasmid–based approach–recapitulate the disease phenotype and generate significantly fewer motor neurons at later developmental time periods in culture compared to two separate control subject iPSC lines. During motor neuron development, both SMA lines showed an increase in Fas ligand-mediated apoptosis and increased caspase-8 and-3 activation. Importantly, this could be mitigated by addition of either a Fas blocking antibody or a caspase-3 inhibitor. Together, these data further validate this human stem cell model of SMA, suggesting that specific inhibitors of apoptotic pathways may be beneficial for patients.

## Introduction

Spinal muscular atrophy (SMA) is a recessively inherited pediatric neuromuscular disease characterized by degeneration of spinal motor neurons, resulting in progressive muscle wasting, paralysis, and often death [1]. Depending on the age of onset and clinical symptoms, the disease is classified into four types (Type I–IV). SMA is caused by a deletion or mutation in the survival motor neuron 1 (*SMN1*) gene, which is responsible for the production of a ubiquitously expressed SMN protein [2], [3]. SMN protein is known to be important in pre-messenger RNA splicing, processing and in small nuclear ribonucleoprotein (SnRNP) biogenesis [4], [5], but it remains unclear why motor neurons (MNs) are particularly vulnerable [6]. Humans have a second, almost identical copy of *SMN1*, termed *SMN2*. *SMN2* has a single nucleotide C to T transition that leads to alternative splicing and removal of exon 7 rendering the majority of the protein produced unstable and non-functional [7]. However, ∼15% of SMN protein derived from *SMN2* is functional, and it has been shown that patients with more copies of *SMN2* have decreased disease severity [8]. As such, drug development strategies have targeted *SMN2* for therapeutic intervention [9]–[12].

The neuronal apoptosis inhibitor protein (*NAIP*), found on chromosome 5 in close proximity to *SMN1*, is mutated in greater than half of all SMA type 1 cases [13] and loss of *SMN1* itself may also lead to motor neuron cell death through apoptosis [14], [15]. While it has been shown that SMN on its own has minimal anti-apoptotic effects, a significant reduction in both Fas-mediated and Bax-mediated apoptosis was observed through direct interaction with the anti-apoptotic factor Bcl-2 [16]. However, the interaction of Bcl-2 and SMN is contentious, as another study clearly showed that SMN and Bcl-2 do not directly interact *in vivo* and suggested that overexpression of these proteins *in vitro* may have resulted in aggregation artifacts in the Iwahashi et al. study [17]. Although the exon 7 deleted form of SMN extends the life of “severe” SMA mice [18], it has been shown not to have a direct anti-apoptotic benefit, thus providing a possible explanation as to why *SMN2* does not prevent the apoptotic process [16], [19]. Furthermore, other reports have demonstrated an increase in apoptosis and aberrant motor neuron growth in the absence of SMN in SMA animal models and *in vitro* [20]–[23]. Importantly, motor neuron protection was conferred after anti-apoptotic intervention in these models.

Although there are several transgenic mouse models of SMA available [24]–[26], the fundamental biological differences between mice and humans will always be a barrier to translating these studies to human clinical trials. Thus the generation of iPSCs from adult human fibroblasts [27]–[29] is opening up a complementary field of human disease modeling for neurological diseases [30]. We have previously shown that iPSCs derived from a Type I SMA patient showed a selective loss of MNs over time in culture [31]. Therefore, in the current study, we sought to investigate the pivotal cell death signaling mechanisms leading to fewer MNs, using the original patient line and a newly generated virus-free line from a second Type I SMA patient. We demonstrate that both SMA patient-iPSC lines reproducibly have diminished MN numbers in temporal manner. Furthermore, we show that this phenotype observed in SMA MN cultures involves the activation of Fas ligand-mediated apoptotic pathway via caspases-8 and -3. Finally, blocking this pathway prevents MN cell death suggesting that this pharmacological approach may be important for patients with this disease.

## Results

### Generation of a Virus-free iPSC Line from a Second SMA Patient

**Figure 1:**
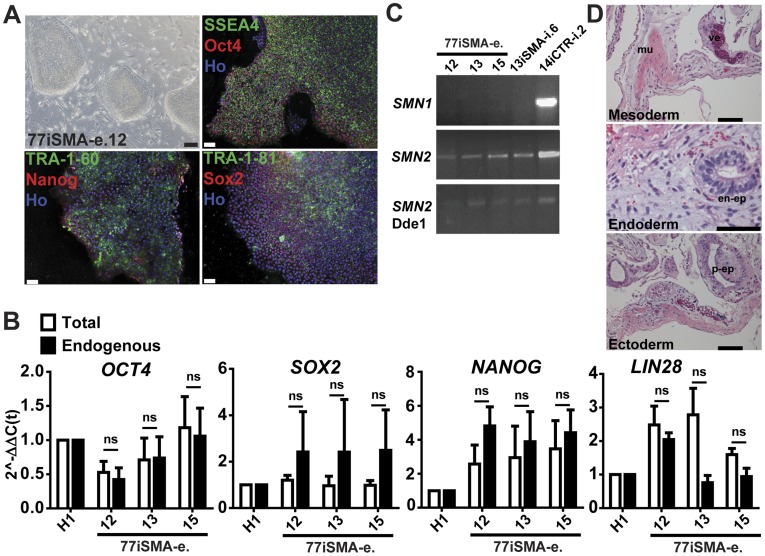
Generation and characterization of a new SMA-iPSC line. (**A**) Bright field images of the 77iSMA-e.12 SMA line showing typical pluripotent stem cell colony morphology, made by a combination of episomal vectors. Immunostaining shows expression of embryonic stem cell surface antigen SSEA4, Tra-1-60, Tra-1-81, and nuclear Oct4. (**B**) Quantitative RT–PCR analyses of *OCT4, SOX2, NANOG, cMYC, KLF4, LIN28* expression in seven clones of 77iSMA iPSCs relative to H1 hESCs. “Endogenous” indicates that primers were included in the 3′ untranslated region (UTR) measure expression of the endogenous gene only, whereas “total” indicates that primers in coding regions measure expression of both the endogenous gene and the transgene if present**.** Gene expression differences were not significant (ns) by one-way ANOVA and data are represented as mean ± SD. (**C**) 77iSMA and 13iSMA iPSCs show the expected lack of *SMN1* expression and maintenance of *SMN2* expression. (**D**) Hematoxylin and eosin (HE) histology from teratoma tissue in nude mice kidney capsule grown for 6 weeks, showing striated muscle (mu) and a vessel (ve) of mesodermal origin, endodermal-epithelia (en-ep) of intestinal character, and ectodermal epithelia of peridermal (p-ep) character. Scale bars: 100 µm.

With a large number of patient lines and subsequent clones being generated by a number of consortia and other groups, the iPSC lines reported here will follow the naming convention recently suggested [32] **.** In our initial report, we generated iPSCs from fibroblasts of a type 1 spinal muscular atrophy (SMA1) patient (Coriell repository identifier: GM038**13**; iPSC line UW13iSMA-i.6, referred here as 13iSMA) and his unaffected mother (GM038**14**; iPSC line UW14iCTR-i.2, referred here as 14iCTR) [31]. In order to reproduce the phenotype seen in an independent line, we generated a second iPSC line from another SMA1 patient (23 month old) fibroblast sample (GM096**77**; iPSC line UW77iSMA-e.x, referred here as 77iSMA) by a single nucleofection with episomally expressed oriP/EBNA1-based pluripotency plasmids in combination with small molecules [33]. Seven clones were generated (Fig. 1, Fig. S1A) and have been validated for pluripotency markers including expression of cell surface (SSEA4, TRA-1-60 and TRA-1-81) and nuclear (Oct4, Nanog and Sox2) proteins (Fig. 1A, Fig. S1B). Using qRT-PCR analyses, we failed to detect any residual transgene expression in the 77iSMA iPSC clones as no significant differences were observed between total and endogenous gene expression (Fig. 1B, Fig. S1C). The absence of SMN1 and the maintenance of SMN2 expression in the SMA-iPSCs were also confirmed using PCR (Fig. 1C). Clone 77iSMA.e12 produced a teratoma after 6 weeks in SCID mice (Fig. 1D) and was therefore selected for further examination and subsequently referred to as 77iSMA. We also included a separate control iPSC line (GM021**83**; iPSC line UW83iCTR-i.8, referred here as 83iCTR [34]) for analysis in this study, which was characterized using the standard battery of pluripotency assays (Fig. S2A–C).

### Motor Neurons can be Produced from Control and SMA Patient iPSCs

**Figure 2:**
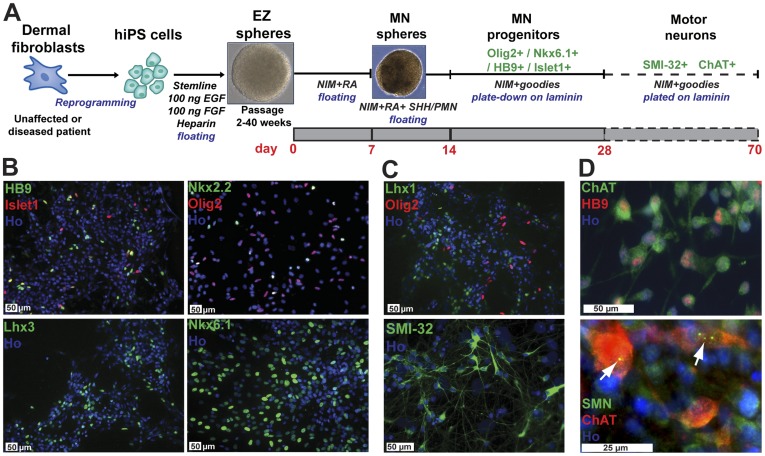
Motor neuron (MN) differentiation from iPSCs. (**A**) Schematic representation of MN differentiation from iPSCs out to 10 weeks. (**B, C** and **D**) Differentiated human iPSCs are capable of developing into both (**B**) early, (**C**) intermediate, and (**D**) mature MN markers indicative of lineage restriction. (**D**) Detection of SMN protein in the nuclei (gems) and cytoplasm of choline acetyltransferase (ChAT) positive MNs derived from control iPSCs (83iCTR). Representative images for MN differentiation depicted here are from healthy control subject iPSCs (14iCTR or 83 iCTR). Scale bars: 50 µm.

**Figure 3:**
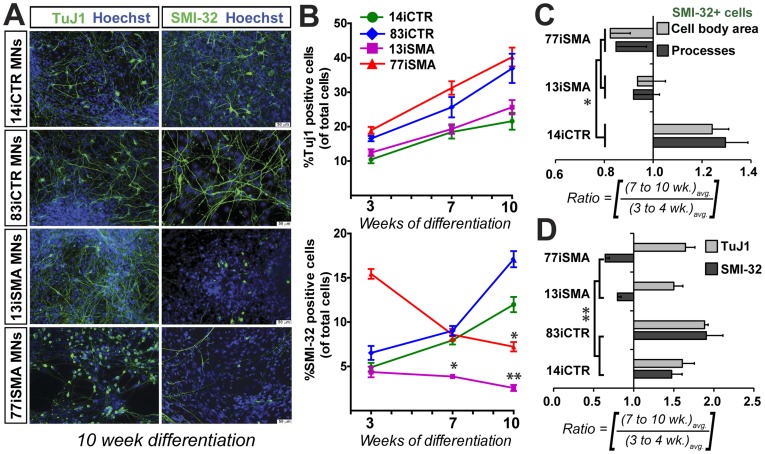
SMA MN cultures display a degenerative phenotype. (**A**) At 10 weeks of differentiation, both SMA patient-iPSC lines show a significant reduction of SMI-32+ MNs compared to both control iPSC lines. However, the Tuj1+ (βIII-tubulin) neuronal population is unaffected. These data are represented as mean ± SEM and are quantified in (**B** and **C**); the graphs are represented as percent positive TuJ1 or SMI-32 cells of the total Hoechst positive population. There was no significant difference in Tuj1 positive neuron numbers observed between the control and SMA cells at 3, 7 and 10 weeks of MN patterning. (**C**) There is a reduction of total cell body area and total number of processes in the SMA cell lines at late stages of differentiation compared to control iPSCs. (**D**) Meta-analysis of SMI-32 and TuJ1 counts confirms a significant reduction in SMI-32+ MNs in SMA cells. (**C** and **D**) Data are represented according to the longitudinal differentiation equation (*Materials and Methods*) as mean ± SEM, n = 4 independent experiments. Scale bars  = 50 µm. * p<0.05, ** p<0.01.

We developed a simple method to generate multipotent neural stem cells (NSCs) from human iPSCs without utilizing embryoid body formation (Fig. 2A). The iPSC colonies were lifted from mouse embryonic fibroblast feeder layers and cultured in suspension medium containing high concentrations of EGF (100 ng/ml) and bFGF (100 ng/ml) supplemented with heparin. Spherical aggregates of NSCs form within 1 week from both the SMA patient and unaffected control iPSC colonies and could be expanded for over 40 weeks in culture, termed NSCs^EFH^. These NSCs could be driven towards motor neuron (MN) fate by first caudalizing with all-trans retinoic acid (ATRA) and then ventralizing with purmorphamine (PMN; Smoothened agonist in the Hedgehog signaling pathway; [35]). Emerging MNs were characterized by immunocytochemistry for early MN markers, Olig2, HB9, Islet-1, Nkx2.2, Nkx6.1, and Lhx3 (Fig. 2B), and later expression of intermediate/mature MN markers, Lhx1, SMI-32 (a 200 kDa neurofilament protein) and choline acetyltransferase (ChAT) (Fig. 2C,D). Further, co-expression of HB9/ChAT confirmed regional specification (Fig. 2D). Finally, we were able to identify nuclear gems and cytoplasmic SMN in ChAT positive neurons differentiated from control 83iCTR cell line (Fig. 2D), whereas SMA-iPSCs maintained their characteristic loss of SMN protein over time in differentiation (Fig. S3). All four iPSC lines were capable of generating significant numbers of MNs at 3 weeks of differentiation (Fig. 3B).

### SMA Patient-derived Motor Neurons Degenerate Over Time

We previously reported that following robust MN production at 4 weeks, there was then a significant decline in MNs over time in the SMA cultures [31]. In agreement with this previous study, we again observed significantly fewer MNs over time following ten weeks of differentiation for both the 13iSMA (2.6%) and 77iSMA (7.2%) cultures when compared to controls 14iCTR (11.9%) and 83iCTR (17.1%) (Fig. 3A). Interestingly, while the total number of neurons was similar between the lines, the new 77iSMA line actually had significantly more MNs at early time points (Fig. 3B), probably reflecting inter-line variability. However, the longitudinal analyses revealed that while all cultures continued to undergo neurogenesis at similar levels over time, and the control cultures continued to produce motor neurons between 3–10 weeks, the SMA cultures were found to contain significantly fewer MNs (p<0.01; Fig. 3B). In addition to a decline in MN numbers and/or lack of production, there was also a significant reduction in the total cell body area over time (0.82–0.94 fold change, p<0.05) and number of processes (0.85–0.92 fold change, p<0.05) of SMI-32+ cells in both SMA lines when compared to the control line, which increased 1.3 fold (Fig. 3C). This single representative experiment allowed a direct comparison between the different iPSC lines plated at the same time, density and identical passage numbers. We next tested the robustness of the model across multiple experiments and different iPSC lines. Clearly there is some inter-line and inter-experiment variability chiefly due to deviations in exact plating cell densities as a result of clustering of motor neuron precursor aggregates, which subsequently affects the stress levels within the culture system. However, a meta-analysis of four independent MN differentiation experiments revealed that there were significantly fewer SMI-32+ cells at late differentiation time points (8–10 weeks) in the two SMA cell lines (13iSMA and 77iSMA) compared to two control cell lines (14iCTR and 83iCTR), while the TuJ1 numbers constantly increased between 1.5–2 fold over this same time period (Fig. 3D). Taken together, these data confirmed that this model was reliable and reproducible, and agreed with our previously published work [31].

### Apoptotic Markers are Activated in SMA Motor Neuron Cultures

We next wanted to establish whether SMA MNs were dying over time in culture, which has previously also been shown to occur in SMA mouse models [20], [21]. MN cultures derived from SMA and control lines were first fixed and stained with Hoechst 33528 to detect nuclear condensation, a hallmark of apoptosis [36]. The percentage of apoptotic cells at 7–10 weeks of differentiation was significantly greater in the 13iSMA (12.6%) and 77iSMA (13.1%) iPSC motor neuron cultures compared to the control iPSCs MN cultures, 14iCTR (7.7%) and 83iCTR (8.2%) (Fig. S4), suggesting that SMA-iPSC MN cultures are undergoing active apoptosis.

**Figure 4:**
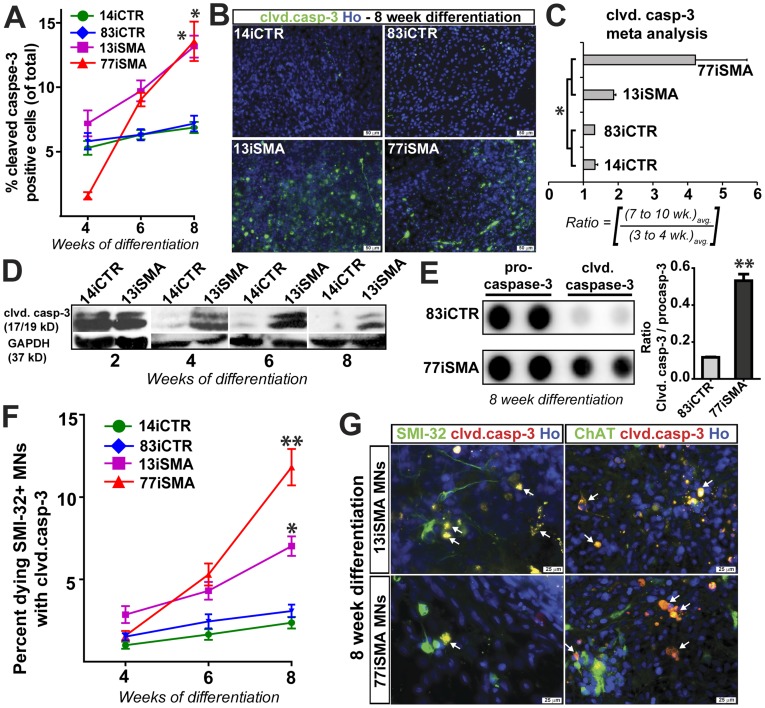
Detection of apoptosis in SMA MN cultures. (**A** and **B**) Both SMA-iPSC MN cultures showed an increase in cleaved caspase-3 staining over time compared to control iPSC MN cultures. Representative images are shown in *B*. (**C**) Meta-analysis confirms the increase in caspase-3 activation in SMA-iPSC MN cultures. Data are represented according to the longitudinal differentiation equation (*Materials and Methods*) as mean ± SEM, * p<0.05, n = 4 independent experiments. (**D** and **E**) Western blot analysis of cell lysates from SMA-iPSC MN cultures shows significant and sustained activation of caspase-3 compared to control iPSCs. (**F** and **G**) There were significantly more cleaved caspase-3/SMI-32 (**F**) and cleaved caspase-3/ChAT (**G**) double positive cells in both SMA-iPSC lines compared to control iPSC lines over time. Data in (**F**) are represented as mean ± SEM. White arrows indicate doubled labeled SMI-32 MNs also positive for cleaved caspase-3 and the 13iSMA and 77iSMA cultures Scale bars: 50 µm (**B**) and 25 µm (**G**).

Apoptosis is known to be executed by the cleavage of pro-caspases, such as initiators (caspase-8 and caspase-9) and executioners (caspase-3 and caspase-7), to their active cleaved forms. We first examined whether the final executioner caspase-3 was involved in SMA specific MN cell death using an antibody specific to its cleaved active form. In agreement with the nuclear condensation assay, a representative experiment exhibits significantly more active caspase-3 positive cells at 8 weeks of differentiation in the 13iSMA (13.2%) and 77iSMA (13.6%) iPSC cultures compared to the 14iCTR (6.8%) and 83iCTR (7.1%) cultures (Fig. 4A,B). A further meta-analysis over four independent MN differentiation experiments also showed a significant increase in cleaved caspase-3 positive cells at late differentiation time points (8–10 weeks) in the two SMA cell lines when compared to controls (Fig. 4C). Immunoblot analysis confirmed this result revealing higher levels of cleaved caspase-3 isoforms (17/19 kDa) in the 13iSMA-iPSC MN cultures (Fig. 4D,E). This result was further substantiated by a significant increase in the ratio of cleaved caspase-3 to procaspase-3 in 8 week old 77iSMA MN cultures using a human apoptosis proteome profiler array (Fig. 4E). We next asked whether apoptosis was occurring in the developing MNs by double labeling with antibodies to cleaved caspase-3 and SMI-32. Significantly more double positive MNs were observed in the SMA cell lines (6.9% for 13iSMA (p<0.05) and 10.9% for 77iSMA (p<0.01)) compared to the control lines (2.3% for 14iCTR and 3.3% for 83iCTR) (Fig. 4F,G). Very similar results were seen with double labeling of cleaved caspase-3 with ChAT (Fig. 4G). Taken together, these data show that there is enhanced apoptosis in the SMA lines.

### SMA Motor Neuron Cultures Show Increased Caspase-8 Activation and Levels of Fas Ligand

**Figure 5:**
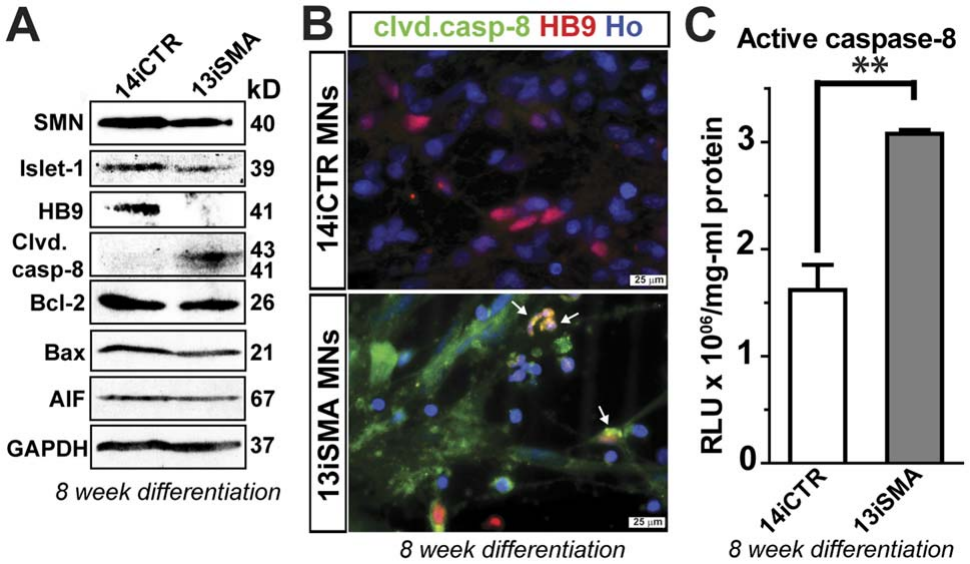
Activation of caspase-8 in SMA MN cultures. (**A**) Western blot analysis of MN patterned SMA-iPSCs cell lysates at 8 weeks show reduced SMN and MN markers Islet-1 and HB9, but increased activation of caspase-8 compared to control iPSCs. There was no difference in expression levels of Bax, Bcl-2, and AIF. GAPDH was used as a loading control. (**B**) Immunocytochemistry detected an increase in cleaved caspase-8 in 13iSMA MN cultures**.** White arrows indicate doubled labeled HB9 positive MNs that are also positive for cleaved caspase-8. (**C**) An increase in caspase-8 activation in 13iSMA MNs was confirmed with the Caspase Glo-8 assay, measuring caspase-8 activity by release of luminescence upon activation of caspase-8 and a cleavage of a target peptide. Data are represented as mean ± SEM, n = 3 experiments.

**Figure 6:**
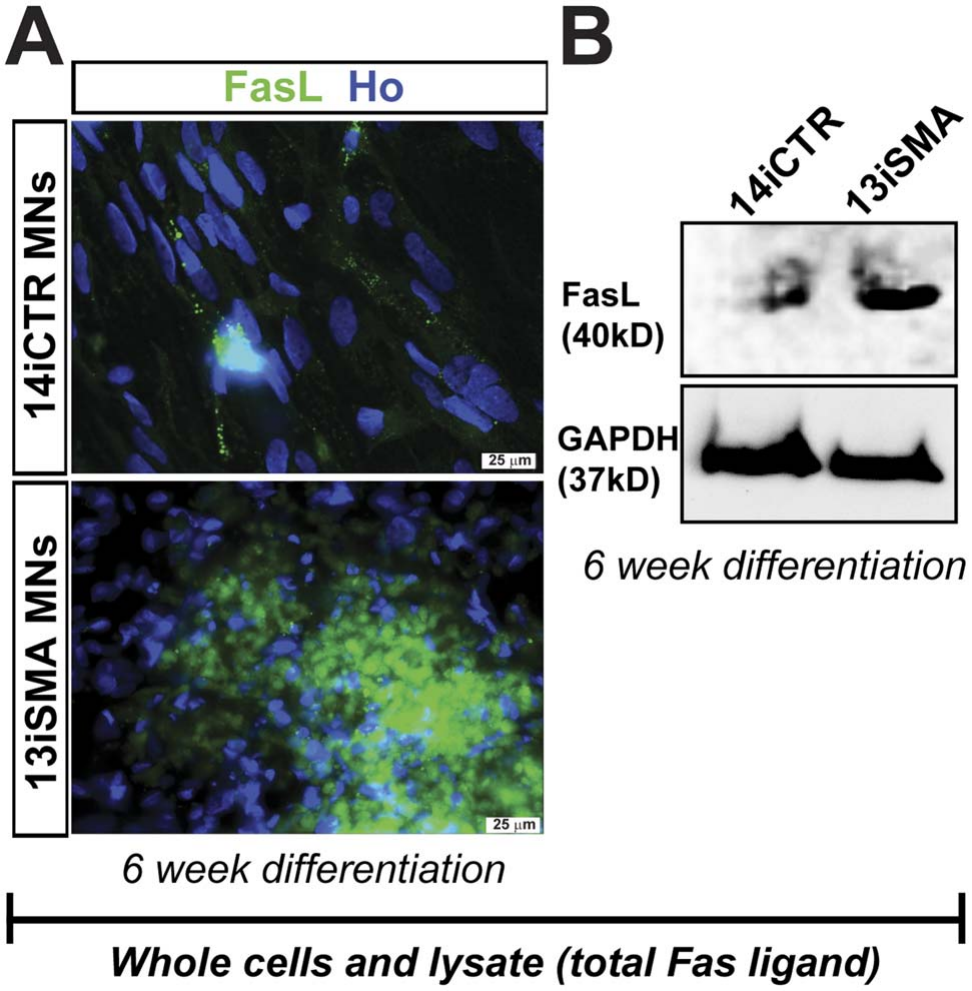
Fas ligand over-expression in SMA MN cultures. (**A** and **B**) Expression of total membrane-bound Fas ligand was increased in 13iSMA cells after 6 week differentiation seen by (**A**) immunocytochemistry and (**B**) Western blot analysis. Data shown here are representative of n = 3 independent experiments.

We next explored which apoptotic pathways led to the expression of cleaved caspase-3 in SMA. We did not observe changes in the levels of mitochondrial-associated apoptosis signaling molecules including Bcl-2, Bax, cleaved caspase-9, and AIF (Fig. 5A and data not shown). However, using western blot analysis we identified an increase in activation of initiator procaspase-8 to its cleaved form in the SMA-iPSC MN cultures compared to control (Fig. 5A). This was also confirmed by immunocytochemistry (Fig. 5B) and luminescence activity (Fig. 5C) in 13iSMA MN cultures. Greater numbers of HB9/cleaved caspase-8 positive MNs were also observed in 13iSMA MNs compared to the 14iCTR MNs (Fig. 5B). Caspase-8 is an important player in the apoptotic pathway that is triggered by the binding of ligands (e.g. Fas ligand (FasL), a TNF family transmembrane protein) to their receptors leading to the recruitment of adaptor proteins [37]. As such, we observed increased levels of membrane-bound Fas ligand (mFasL) by immunocytochemistry (Fig. 6A) and immunoblotting (Fig. 6B *)* in the SMA patient MN cultures at 6 weeks of differentiation. Collectively, these results show that apoptotic cell death in the SMA MN cultures is mediated through FasL, caspases-8 and -3 signaling.

### Motor Neurons are Rescued by Blocking Apoptosis in SMA MN Cultures

**Figure 7:**
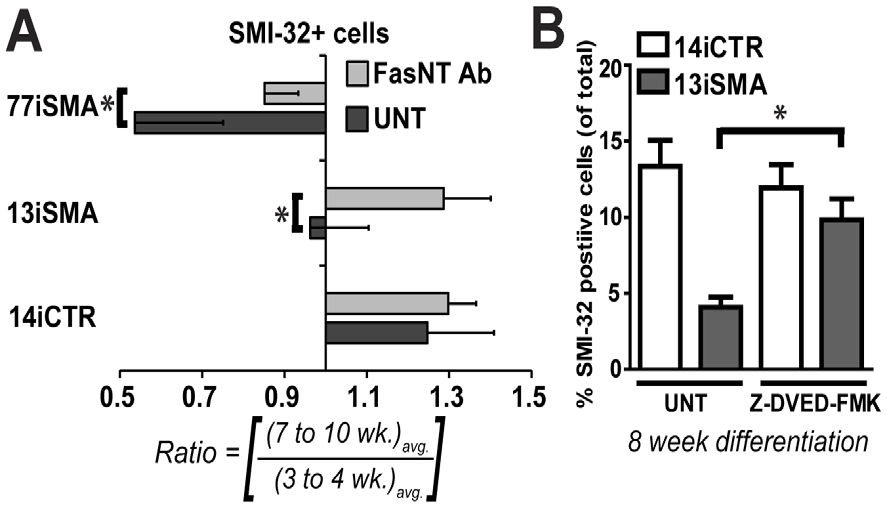
Rescuing motor neuron loss in SMA cell lines. (**A**) Treatment with Fas neutralizing antibody (FasNT Ab) significantly protected 77iSMA and 13iSMA SMI-32+ motor neurons at 8 weeks relative to 4 weeks. Data are expressed according to the longitudinal differentiation equation (*Materials and Methods*) as mean ± SEM, * p<0.05, n = 2 independent experiments. (**B**) Treatment of 13iSMA MN cultures with the specific caspase-3 inhibitor Z-DVED-FMK significantly protected SMI-32+ MNs at 8 weeks of differentiation, * p<0.05, n = 2 independent experiments.

In order to establish whether inhibiting apoptotic pathways could rescue the diminished MN numbers in the SMA lines, we first utilized the antagonistic ZB4 clone of anti-Fas monoclonal antibody (FasNT Ab), previously shown to block the apoptosis-inducing activity mediated through the Fas receptor pathway [38]. Treatment with FasNT Ab beginning at week 2 and maintained for the duration of the differentiation process significantly increased MN number in SMA-iPSC cultures at 8 weeks of differentiation (33% for 13iSMA and 31% for 77iSMA, p<0.05 (Fig. 7A). Similarly, culturing 13iSMA MNs in the presence of the caspase-3 specific inhibitor Z-DVED-FMK starting at week 3 significantly rescued the SMI-32+ cell population to the level of the 14iCTR (Fig. 7B). Collectively, these data show that the diminished MN numbers in SMA patient-iPSC lines is dependent upon apoptosis, specifically through the Fas-mediated pathway.

## Discussion

The molecular mechanisms that lead to the development of the SMA pathology are unclear. For this reason, despite substantial research in the area, an effective treatment for this disease does not yet exist. As such, there is a need to identify therapeutic strategies that delay the advance of SMA pathology. Following lineage restriction of hiPSCs to generate motor neurons, previously shown to be functional [31] and electrophysiologically active [39], we identified molecular markers of apoptosis in SMA-iPSC MN cultures. In the present study, we demonstrate using two independent SMA and two control iPSC lines that there were significantly fewer MNs at 10 weeks of differentiation from SMA patient-iPSCs. Importantly, we show here that this phenotype could be rescued by blocking the Fas receptor or inhibiting caspase-3. As such, our data suggest that apoptosis plays an important role in disease progression, and therapies targeting this cascade may have important clinical applications.

The lentiviral SMA type 1 line (13iSMA) [31] used in this study produces similar number of SMI-32+ MNs compared to two control lines at early time points in culture, while the new virus-free SMA type I line (77iSMA) initially produces far greater MNs than all the lines combined. This would suggest that in this SMA stem cell model, the patient iPSC lines are competent in generating MN progenitors at early stages of differentiation and argue against a serious fetal developmental maturation error as suggested in a recent study examining post-mortem pathological analyses from spinal cords of SMA patients [40]. Between 3 and 10 weeks of differentiation, the SMA cultures show a decline in the total number of MNs in comparison to the control cultures (Fig. 3B). This could either represent death of the motor neurons over time, or failure to increase in number due to deficits in motor neuron progenitor pools. However, we show here that greater numbers of MNs in the SMA cultures were double labeled with caspase-3 (Fig. 4F,G) when compared to control cultures, suggesting that at least a proportion of MNs were actively undergoing apoptosis.

We have observed that there is inter-line variation in the propensity of hiPSCs and hESCs towards terminal MN differentiation, which is likely due to the intrinsic characteristics of the lines. This observation is confirmed by a recently published study where 16 iPSC lines were tested by independent labs for MN production using standard protocols [41]. Although all cell lines were capable of generating MNs, significant and reproducible quantitative differences were revealed in the propensity of the lines for terminal differentiation. This cautions against the direct comparison of cell numbers at any stage – pluripotent stem cells, progenitors or terminally differentiated derivatives – between control and patient iPSC lines for a reproducible disease phenotype. Therefore, in cases where a disease phenotype is expected in differentiated cells, we suggest that validation of a disease phenotype should be performed by a temporal, longitudinal study collecting data at multiple time points for an intra- and inter-line comparison. Using this methodology, each cell line serves as its own control with regard to starting and ending numbers of specific types of neurons or other cell types. Here we show that MNs generated from SMA patients undergo selective degeneration in a temporal manner, and that this is associated with the activation of the Fas-mediated apoptosis. Our findings of MN degeneration in SMA-iPSC lines are also confirmed by a very recent report which shows that five clonal iPSC lines from a single SMA patient (GM09677 patient fibroblasts were used in this report analogous to the source of the 77iSMA line used in our study), made using retroviral integrating vectors, exhibit a reduced capacity to form MNs and abnormalities in neurite outgrowth [42], all of which could be rescued by ectopic expression of SMN.

The discovery that the neuronal apoptosis inhibitor protein (NAIP) on chromosome 5 was mutated in greater than half of all SMA type 1 cases [13] led to early suggestions that apoptosis may be directly involved with SMA. Interestingly, deletions in the NAIP gene in the same patients may increase the severity of disease and thus act as a modifier [14], [15]. Furthermore, while the major function of SMN is the biogenesis of spliceosomal snRNPs, it has also been shown to modulate apoptosis [16]. Further support for involvement of apoptosis in SMA comes from a number of *in vitro* and *in vivo* studies [16], [19]–[23], [43]. Our data showing significant increases in active caspase-3 within the differentiating SMA MN cultures lends further direct support to the idea of apoptosis in the human disease. This is in contrast to a recent report from Ito and colleagues in which no significant difference in caspase-3 activation was observed between SMA and control fetal post-mortem spinal cord tissue samples [40]. This could be due to a variety of reasons including timing of analysis, tissue processing, methods of detection, and intrinsic differences between *in vitro* and *in vivo* studies. Nevertheless, apoptotic signals are activated in our *in vitro* model and warrant further investigation in SMA pathophysiology.

Clearly apoptosis is a complex process where one of the major pathways involves binding of ligands, such as TNF or FasL, to their death receptors (TNFR or FasR, respectively), leading to the recruitment of adaptor proteins and the subsequent activation of caspase-8 [44]. Downstream activation of caspase-3 leads to cleavage of substrates vital to cell function. Here we show that cells within MN cultures of SMA patient-iPSCs showed significant chromatin condensation as well as activation of initiator caspase-8 and executioner caspase-3. While we cannot eliminate some involvement of mitochondrial-dependent pathways as previously found in a mouse model of SMA [45], the major component appears to be death receptor-mediated. Membrane-bound FasL is a potent activator of the death-receptor mediated apoptosis, and we showed increases in membrane-bound FasL in the SMA samples corresponding to the time of significant MN loss (Fig. 6). Blocking this pathway during differentiation with Fas neutralizing antibody rescues the MNs, further supporting a role for this pathway being active in this disease. SMN levels do not vary temporally in this culture system over the course of MN differentiation in SMA-iPSC lines (Fig. S3), but the current data do not identify a mechanism linking diminished SMN protein to up-regulated Fas ligand. Therefore, further studies will address FasL processing, splicing, transport, and translation specifically in relation to reduced SMN.

During development, apoptosis plays a critical role in neuron pruning such that 50% of MNs initially generated in the spinal cord die [46]. Target tissues play an important role in providing necessary trophic support to maintain MNs. However, embryonic MNs have been shown to activate apoptosis through binding of FasL and activation of caspase-3 independent of trophic factor support [47]. Caspase-3 activation has been observed in healthy control and SMA patient fetal post-mortem spinal cord tissue [40]. Similarly, we also observe caspase-3 activation in the MN cultures from the control and SMA lines at early time points (Fig. 4D), which is presumably due to active apoptotic signals upon the transition from a pluripotent stem cell state to a differentiated state, reflecting non-MN and MN death. If MNs are naturally primed to undergo apoptosis during development, perhaps the lack of SMN in SMA-iPSCs maintains a hyperactive apoptotic process via prolonged caspase-3 activation, and may also explain the disease phenotype observed in patients and experimental models. Alternatively, the MNs in our culture system may be lacking the appropriate target tissue or trophic support. Notably, blocking caspase-3 activation with a commercial inhibitor rescues the MN degeneration phenotype in SMA, signifying the importance of this pathway in our disease model.

The iPSC-derived cultures in this study contain a mixed population of cell types, including ChAT positive motor neurons, Tuj1 positive neurons, and GFAP positive astrocytes (Fig. S5). At this time, technical complexities, such as purification and survival of mature MNs from iPSCs by sorting or panning, make it difficult to distinguish whether a cell autonomous or a non-cell autonomous apoptotic process is involved. The non-neuronal cells play a determinant function in the vulnerability of the neurons in several pathological conditions [48]. Astrocytes, for example, have been shown to dramatically alter the health and survival of MNs in transgenic models of ALS [49] and FasL is known to promote astrocyte reactivity and production of proinflammatory cytokines [50], [51]. Since SMN1 is absent in all cell types, the possibility remains that astrocytes may themselves be dysfunctional and contributing to the apoptotic process. Future studies targeting the HB9 motor neuron-specific promoter in iPSCs in conjunction with co-culture assays will address this issue. The protective effect of FasL antagonistic antibody may occur though an indirect mechanism implicating surrounding cells in our MN cultures.

During the past several years, our understanding of the mechanisms mediating cell death in neurologic diseases has improved considerably. The fact that activation of these pathways is a feature of a broad range of neurologic diseases makes them important and attractive therapeutic targets. Therefore, conferring neuroprotection by interrupting apoptosis and preserving mitochondrial integrity are being actively explored by companies and academics. For example, monoamine oxidase inhibitors, selegiline and rasagiline, in Alzheimer’s and Parkinson’s disease [52], and dexpramipexole, a lower affinity non-ergot dopaminergic autoreceptor agonist, for the potential treatment of amyotrophic lateral sclerosis [53] are being investigated. Additionally, combination therapies targeting apoptosis with other pathways are being tested for Parkinson’s and Alzheimer’s disease including antioxidants, cell cycle inhibitors, JNK inhibitors, GSK3β inhibitors, and STATINS [54], which could also be suitable strategy for delaying the progression of in SMA. Despite the lack of information from clinical trials, natural product compounds such as resveratrol and melatonin are attracting considerable attention because of their antioxidant and anti-apoptotic action in addition to low toxicity in humans [55], [56]. Resveratrol has also been shown to increase full length SMN transcript and protein in SMA fibroblasts [57]. The data presented here further suggest that apoptotic pathway inhibition may be a therapeutically relevant target for SMA. Furthermore, by scaling up the current system for high content screening studies [58], it may be possible to test these novel compounds for efficacy in this novel human model prior to administration to patients, and thus increase the possible chances of success.

## Materials and Methods

### Ethics Statement

Human fibroblast cell lines were obtained from the Coriell Institute for Medical Research. The Coriell Cell Repository maintains the consent and privacy of the donor fibroblast samples. All the cell lines and protocols in the present study were carried out in accordance with the guidelines approved by institutional review boards at the University of Wisconsin-Madison, Cedars-Sinai Medical Center, and Medical College of Wisconsin. All the animal care treatment protocols and procedures in the present study were carried out in accordance with the guidelines approved by the Cedars-Sinai Institutional Animal Care and Use Committee (IACUC) and National Institutes of Health standards of animal care.

### Virus-Free Cellular Reprogramming

Fibroblast cell lines from patients with SMA (13iSMA: GM03813; 77iSMA: GM09677) and normal controls (14iCTR: GM03814; 83iCTR: GM02183) were obtained from the Coriell Institute for Medical Research. Reprogramming of the new 77iSMA line was performed using pEP4-E02S-CK2M-EN2L and pEP4-E02S-ET2K vectors (Addgene, according to previously published protocols [28]). Detailed descriptions of these procedures are provided in Text S1.

### Neural Stem Cell Culture and Motor Neuron Differentiation

Briefly, iPSC colonies were differentiated to make NSCs by gently lifting from MEF feeders in ultra-low attachment flasks with Stemline Neural Expansion Media (Sigma) supplemented with, EGF (100 g/ml), fibroblast growth factor-2 (FGF-2; 100 ng/ml), and heparin (5 µg/ml). NSC spheres stabilized 3 weeks after the first passage following generation and were utilized regularly for further motor neuron (MN) differentiation by placing NSC spheres were placed in neural induction medium (1∶1 DMEM/F12 and 1% N2) in the presence of all-trans retinoic acid (RA; 0.1 µM) for 1 week. This was followed by the addition of purmorphamine (PMN; 1 µM) or Sonic Hedgehog (SHh; 10 ng/ml) for another 1–3 weeks and plate down on poly-ornithine/laminin-coated coverslips to induce maturation to MNs. RNA was isolated and used for gene expression analysis using primers listed in Table S1. For immunostaining and immunoblotting, undifferentiated or differentiated iPSCs were fixed with 4% paraformaldehyde and stained using the antibodies listed in Table S2. Detailed descriptions of these procedures are provided in Text S1.

### Apoptosis Profiler Array

Expression profiles of apoptosis-related proteins (pro- and cleaved-caspase-3) spotted in duplicate on nitrocellulose membranes along with capture antibodies were analyzed using Human Apoptosis Proteome Profiler Array (R & D systems), according to manufacturer’s instructions. Briefly, equal amounts (200 µg) of protein from 8 week differentiated motor neuron cultures lysate were incubated overnight with the membranes, followed by washes at each intermediate step, incubation with detection antibodies, Streptavidin-HRP and chemiluminescent reagents (Pierce) and exposure to X-ray film.

### Apoptosis Inhibitor Treatment

SMA and control iPSCs were treated with either Fas neutralizing antibody (FasNT, clone ZB4, 300 ng/ml) or caspase-3 inhibitor (Z-DVED-FMK, 10 µM, R&D Systems) beginning at 2–3 weeks of MN differentiation through fixation at 8 weeks of differentiation. Fresh inhibitor was added two times a week with each medium change.

### Longitudinal Differentiation Calculations

In order to avoid issues associated with inherent inter-line variations in the propensity of hiPSCs and hESCs to proceed towards terminal neuronal differentiation, we devised a method to perform intra- and inter-line comparison by collecting data at multiple time points for a temporal and longitudinal study. Cell counts were performed and measurements collected at 3, 4, 7, 8, and 10 weeks of differentiation and were put into the following longitudinal differentiation cell ratio formula:
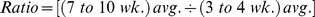



Measurements made at 3 and 4 weeks (i.e. early) or 7 to 10 weeks (i.e. late) were averaged and represented as “(3 *to* 4 *wk.*)*_avg._”* and “(7 *to* 10 *wk.)_avg._”*, respectively. In this case each cell line serves as its own control with respect to starting and ending numbers for motor neurons produced.

### Statistical Analysis

Prizm software (GraphPad software, La Jolla, CA) was used for all statistical analyses. All counting data from immunocyto/histochemical analyses and cell survival were expressed as mean values ± SEM and analyzed by two-tailed *t*-test or two-way ANOVA with Bonferonni *post hoc* test. Differences were considered significant when *p*<0.05.

## Supporting Information

Figure S1 **Characterization of a new virus-free SMA iPSC line.** (**A**) Bright field images of three different clones from 77iSMA show typical pluripotent stem cell colony morphology on irradiated mouse embryonic fibroblasts (MEFs). These lines were made by a combination of two episomal vectors, pEP4-E02S-CK2M-EN2L and pEP4-E02S-ET2K. (**B**) Immunostaining of three clones from 77iSMA iPSCs shows expression of embryonic stem cell surface antigen SSEA3 and nuclear Oct4. (**C**) Quantitative RT–PCR analyses of *OCT4, SOX2, NANOG*, and *LIN28* expression in seven clones of 77iSMA iPSCs relative to H1 ESC. “Endogenous” indicates that primers were included in the 3′ untranslated region measure expression of the endogenous gene only, whereas “total” indicates that primers in coding regions measure expression of both the endogenous gene and the transgene if present (**Table S1**)**.**
(TIF)

Figure S2 **Characterization of a new control iPSC line.** (**A**) Bright field image of one clone different clones from 83iCTR show typical pluripotent stem cell colony morphology on irradiated mouse embryonic fibroblasts (MEFs). This line was generated by a combination of lentiviral constructs expressing *OCT4, SOX2, c-MYC, KLF-4, NANOG,* and *LIN28*. Immunocytochemical staining of embryonic stem cell surface antigens SSEA-4, TRA-1-60 and nuclear Oct4. Scale bars: 50 µm. (**B**) G-band karyotyping showing a normal karyotype of this line. (**C**) Quantitative RT–PCR analyses of *OCT4, SOX2, NANOG, c-MYC, KLF4, LIN28* total and endogenous gene expression in 83iCTR-i.8 clone of relative to H1 hESC (**Table S1**).
(TIF)

Figure S3 **Loss of SMN protein is maintained during differentiation in SMA iPSC motor neuron cultures.** MN cultures from the SMA lines maintain consistent loss of SMN protein during differentiation. Representative Western blots from cell lysates of 13iSMA line harvested at 1, 4 and 8 weeks of differentiation are shown here. Cyclooxygenase IV (COX IV) is used as a housekeeping loading control.
(TIF)

Figure S4 **Apoptotic index of the iPSC motor neuron cultures.** MN cultures from both SMA iPSC lines had significantly more cells exhibiting characteristics of apoptotic nuclei compared to both control iPSC MN cultures. n = 3 experiments.
(TIF)

Figure S5 **Motor neuron cultures are a mixed population of neuronal, glial and non-neural cells.** Glial and neuronal cells are identified in motor neuron differentiating cultures from SMA and CTR iPSCs by immunostaining for (**A**) GFAP positive astrocytes and TuJ1 positive neurons, as well as (**B**) ChAT stained cholinergic neurons can be identified in the cultures. The cell population consists of ∼25–40% non-neural cells. Scale bars: 25 µm.
(TIF)

Table S1 Primer sets for RT-PCR and qRT-PCR. CDR (Tot.) indicates primers that span the coding region of the gene allowing for monitoring of total gene expression, whereas UTR (End.) indicates primers that span the 3′ or 5′ untranslated region of the gene allowing determination of endogenous gene expression.
(DOC)

Table S2 Antibodies used for immunocytochemistry, immunoblotting and apoptosis inhibition.
(DOC)

Text S1 Supporting materials and methods.
(DOC)
