# Supplementary figures and images for: Inhibition of Apoptosis Blocks Human Motor Neuron Cell Death in a Stem Cell Model of Spinal Muscular Atrophy
Source: PLoS One. 2012 Jun 19;7(6):e39113. doi: 10.1371/journal.pone.0039113 (PMC3378532; doi:10.1371/journal.pone.0039113)

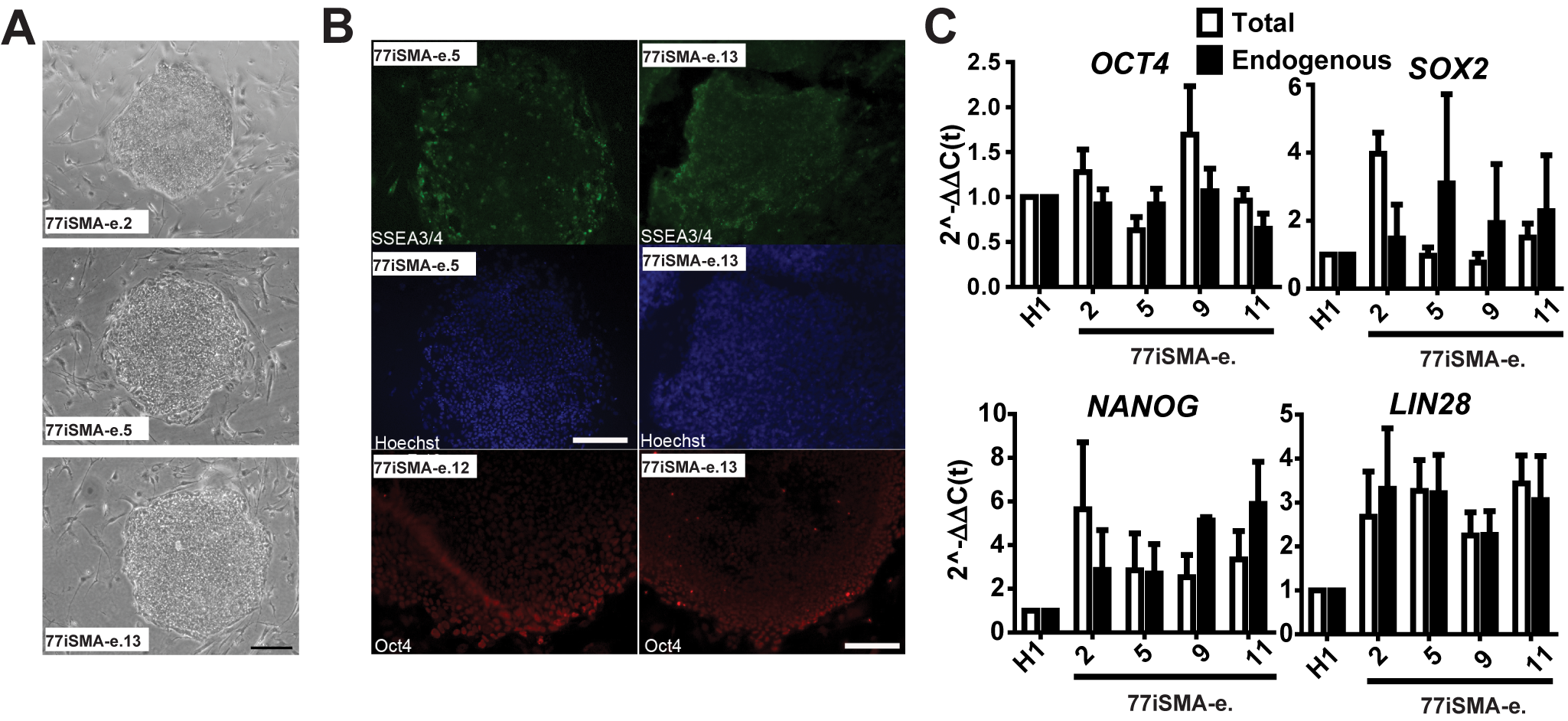

Supplement: Figure S1 — Characterization of a new virus-free SMA iPSC line. (A) Bright field images of three different clones from 77iSMA show typical pluripotent stem cell colony morphology on irradiated mouse embryonic fibroblasts (MEFs). These lines were made by a combination of two episomal vectors, pEP4-E02S-CK2M-EN2L and pEP4-E02S-ET2K. (B) Immunostaining of three clones from 77iSMA iPSCs shows expression of embryonic stem cell surface antigen SSEA3 and nuclear Oct4. (C) Quantitative RT–PCR analyses of OCT4, SOX2, NANOG, and LIN28 expression in seven clones of 77iSMA iPSCs relative to H1 ESC. “Endogenous” indicates that primers were included in the 3′ untranslated region measure expression of the endogenous gene only, whereas “total” indicates that primers in coding regions measure expression of both the endogenous gene and the transgene if present (Table S1). (TIF) [file pone.0039113.s001.tif]

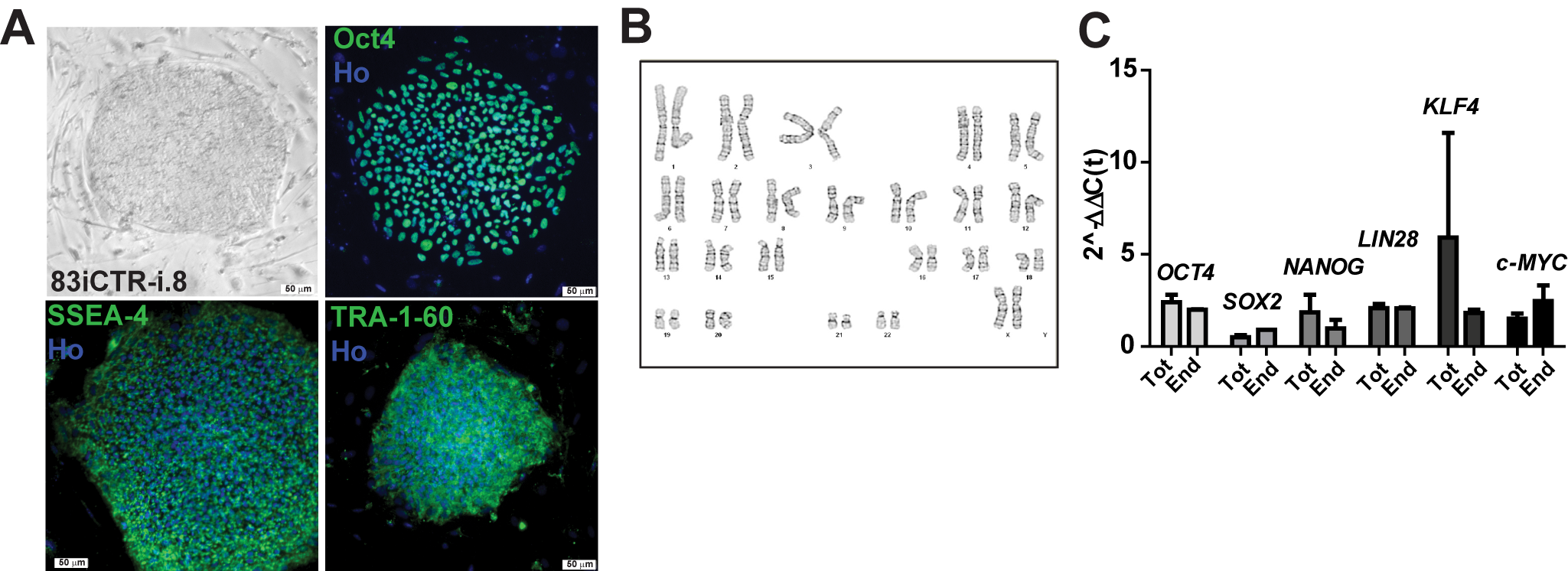

Supplement: Figure S2 — Characterization of a new control iPSC line. (A) Bright field image of one clone different clones from 83iCTR show typical pluripotent stem cell colony morphology on irradiated mouse embryonic fibroblasts (MEFs). This line was generated by a combination of lentiviral constructs expressing OCT4, SOX2, c-MYC, KLF-4, NANOG, and LIN28. Immunocytochemical staining of embryonic stem cell surface antigens SSEA-4, TRA-1-60 and nuclear Oct4. Scale bars: 50 µm. (B) G-band karyotyping showing a normal karyotype of this line. (C) Quantitative RT–PCR analyses of OCT4, SOX2, NANOG, c-MYC, KLF4, LIN28 total and endogenous gene expression in 83iCTR-i.8 clone of relative to H1 hESC (Table S1). (TIF) [file pone.0039113.s002.tif]

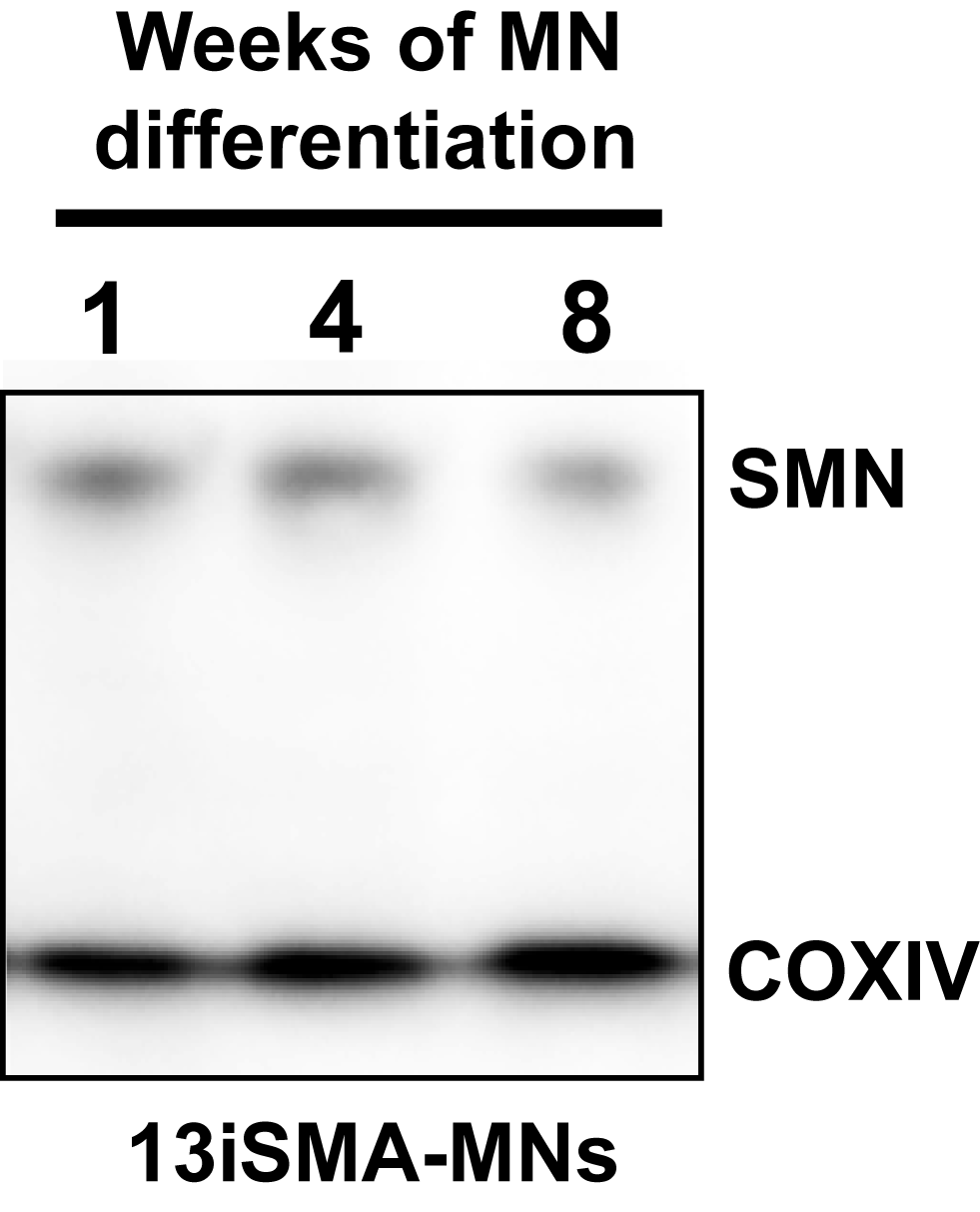

Supplement: Figure S3 — Loss of SMN protein is maintained during differentiation in SMA iPSC motor neuron cultures. MN cultures from the SMA lines maintain consistent loss of SMN protein during differentiation. Representative Western blots from cell lysates of 13iSMA line harvested at 1, 4 and 8 weeks of differentiation are shown here. Cyclooxygenase IV (COX IV) is used as a housekeeping loading control. (TIF) [file pone.0039113.s003.tif]

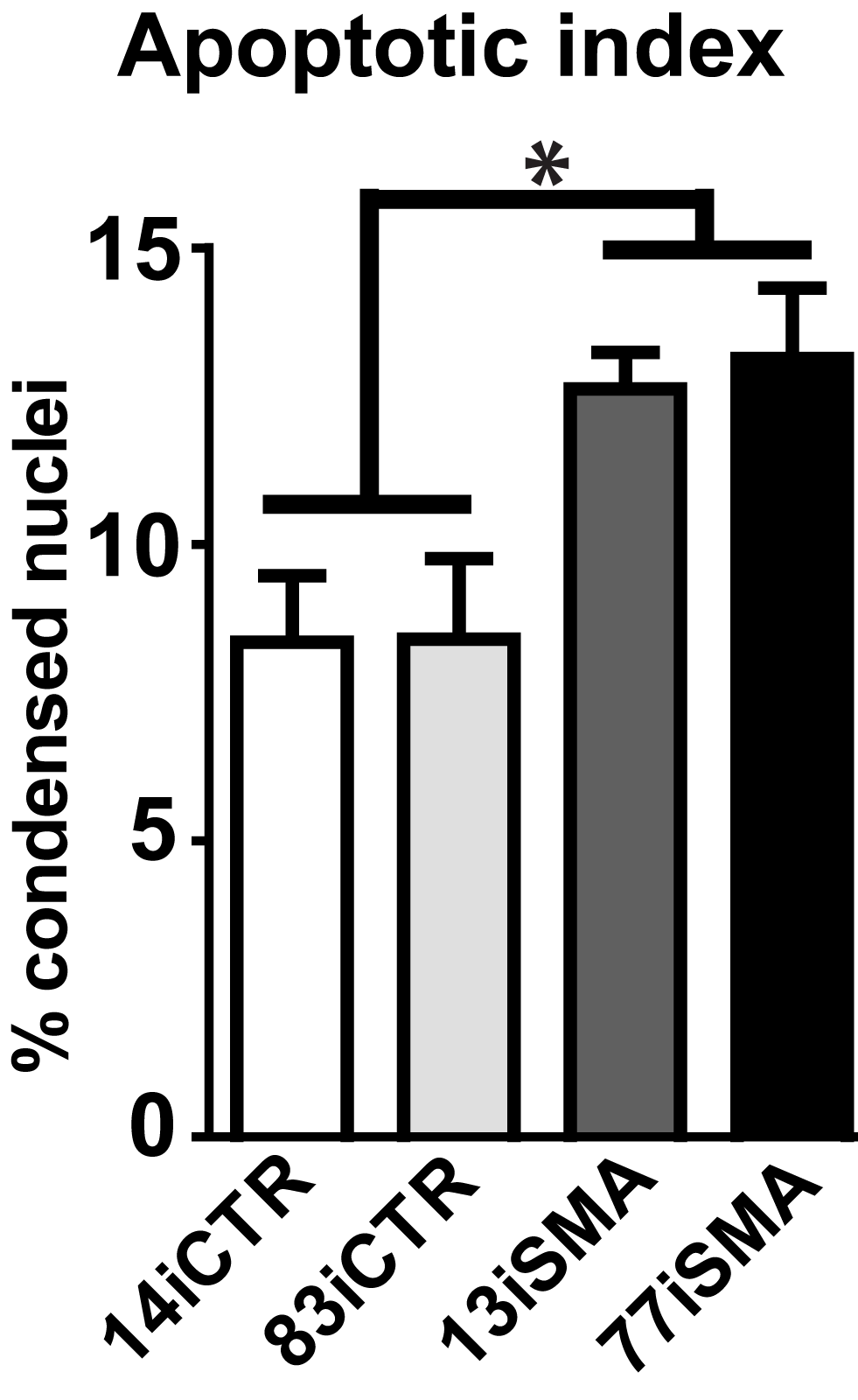

Supplement: Figure S4 — Apoptotic index of the iPSC motor neuron cultures. MN cultures from both SMA iPSC lines had significantly more cells exhibiting characteristics of apoptotic nuclei compared to both control iPSC MN cultures. n = 3 experiments. (TIF) [file pone.0039113.s004.tif]

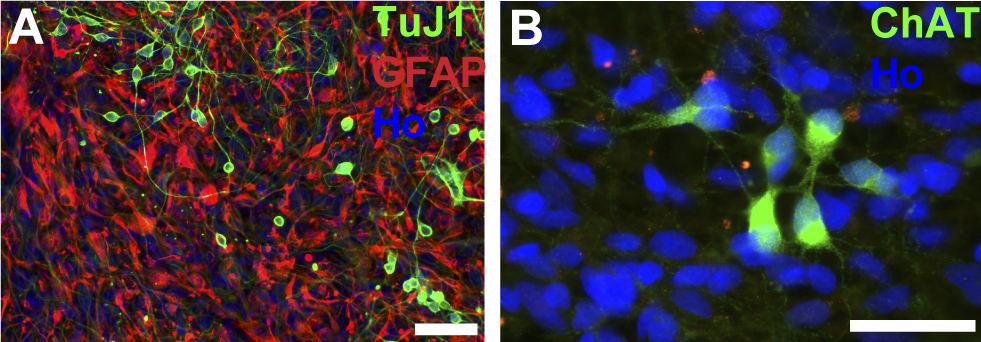

Supplement: Figure S5 — Motor neuron cultures are a mixed population of neuronal, glial and non-neural cells. Glial and neuronal cells are identified in motor neuron differentiating cultures from SMA and CTR iPSCs by immunostaining for (A) GFAP positive astrocytes and TuJ1 positive neurons, as well as (B) ChAT stained cholinergic neurons can be identified in the cultures. The cell population consists of ∼25–40% non-neural cells. Scale bars: 25 µm. (TIF) [file pone.0039113.s005.tif]
