# Supplementary material for: Inhibition of Apoptosis Blocks Human Motor Neuron Cell Death in a Stem Cell Model of Spinal Muscular Atrophy
Source: PLoS One. 2012 Jun 19;7(6):e39113. doi: 10.1371/journal.pone.0039113 (PMC3378532; doi:10.1371/journal.pone.0039113)
Supplement: Table S1 — Primer sets for RT-PCR and qRT-PCR. CDR (Tot.) indicates primers that span the coding region of the gene allowing for monitoring of total gene expression, whereas UTR (End.) indicates primers that span the 3′ or 5′ untranslated region of the gene allowing determination of endogenous gene expression. (DOC) [file pone.0039113.s006.doc]

**Table S1. *Primer sets for RT-PCR and qRT-PCR.***

| **Genes** | **Accession** | **Position** | **Sequences (5’ to 3’)** | |
| --- | --- | --- | --- | --- |
| *GAPDH* | NM_002046 | CDR | Fwd | GTGGACCTGACCTGCCGTCT |
|  |  |  | Rev | GGAGGAGTGGGTGTCGCTGT |
| *OCT4* | NM_002701 | CDR (Tot.) | Fwd | CAGTGCCCGAAACCCACAC |
|  |  |  | Rev | GGAGACCCAGCAGCCTCAAA |
|  |  | 3’UTR (End.) | Fwd | AGTTTGTGCCAGGGTTTTTG |
|  |  |  | Rev | ACTTCACCTTCCCTCCAACC |
| *SOX2* | NM_003106 | CDR (Tot.) | Fwd | TACCTCTTCCTCCCACTCCA |
|  |  |  | Rev | GGTAGTGCTGGGACATGTGA |
|  |  | 3’UTR (End.) | Fwd | AGTCTCCAAGCGACGAAAAA |
|  |  |  | Rev | TTTCACGTTTGCAACTGTCC |
| *NANOG* | NM_024865 | CDR (Tot.) | Fwd | CAGAAGGCCTCAGCACCTAC |
|  |  |  | Rev | ATTGTTCCAGGTCTGGTTGC |
|  |  | 3’UTR (End.) | Fwd | TTTGGAAGCTGCTGGGGAAG |
|  |  |  | Rev | GATGGGAGGAGGGGAGAGGA |
| *LIN28* | NM_024674 | CDR (Tot.) | Fwd | AAGCGCAGATCAAAAGGAGA |
|  |  |  | Rev | CTGATGCTCTGGCAGAAGTG |
|  |  | 3’UTR (End.) | Fwd | AGTGGCCTGGATAGGGAAGT |
|  |  |  | Rev | CTTGGCTCCATGAATCTGGT |
| *c-MYC* | NM_002467 | CDR (Tot.) | Fwd | TCGGAAGGACTATCCTGCTG |
|  |  |  | Rev | GTGTGTTCGCCTCTTGACATT |
|  |  | 5’UTR (End.) | Fwd | TAGGGTGGAAGAGCCGGGCG |
|  |  |  | Rev | GATGCGGCAAGGGTTGCGGA |
| *KLF4* | NM_004235 | CDR (Tot.) | Fwd | GGTGCCCCGAATAACCGCTGG |
|  |  |  | Rev | CTCCGCCGCTCTCCAGGTCT |
|  |  | 3’UTR (End.) | Fwd | ACGATCGTGGCCCCGGAAAAGGACC |
|  |  |  | Rev | TGATTGTAGTGCTTTCTGGCTGGCTCC |
| *SMN1*  *SMN2* | NM_000344  NM_017411 | CDR | Fwd | CTATCATGCTGGCTGCCTCCATTT |
|  |  |  | Rev | ACAATGAACAGCCATGTCCACCAG |

CDR (Tot.) indicates primers that span the coding region of the gene allowing for monitoring of total gene expression, whereas UTR (End.) indicates primers that span the 3’ or 5’ untranslated region of the gene allowing determination of endogenous gene expression.
