# Supplementary material for: Inhibition of Apoptosis Blocks Human Motor Neuron Cell Death in a Stem Cell Model of Spinal Muscular Atrophy
Source: PLoS One. 2012 Jun 19;7(6):e39113. doi: 10.1371/journal.pone.0039113 (PMC3378532; doi:10.1371/journal.pone.0039113)
Supplement: Table S2 — Antibodies used for immunocytochemistry, immunoblotting and apoptosis inhibition. (DOC) [file pone.0039113.s007.doc]

**Table S2. *Antibodies used for immunocytochemistry, immunoblotting and apoptosis inhibition***

| **Antigen** | **Dilution** | **Catalog #** | **Isotype** | **Manufacturer** |
| --- | --- | --- | --- | --- |
| SSEA4 | 1:250 | MAB4304 | mIgG3 | Millipore |
| TRA-1-60 | 1:250 | 09-0010 | mIgM,κ | Stemgent |
| TRA-1-81 | 1:250 | 09-0011 | mIgM,κ | Stemgent |
| OCT4 | 1:250 | 09-0023 | Rabbit IgG | Stemgent |
| NANOG | 1:250 | 09-0020 | Rabbit IgG | Stemgent |
| SOX2 | 1:500 | AB5603 | Rabbit IgG | Millipore |
| HB9 | 1:50 | 81.5C10 | mIgG1, κ | DSHB Iowa |
| ISELT1 | 1:250 | AF1837 | Goat IgG | R & D systems |
| OLIG2 | 1:500 | AB9610 | Rabbit IgG | Millipore |
| NKX2.2 | 1:100 | 74.5A5 | mIgG2b, κ | DSHB Iowa |
| NKX6.1 | 1:100 | F55A10 | mIgG1, κ | DSHB Iowa |
| LHX1 | 1:50 | 4F2 | mIgG1, κ | DSHB Iowa |
| LHX3 | 1:100 | 67.4E12 | mIgG1, κ | DSHB Iowa |
| SMI32 | 1:1000 | SMI-32R | mIgG1 | Covance |
| CHAT | 1:250 | AB144P | Goat IgG | Millipore |
| SMN | 1:250 | 610647 | mIgG1 | BD Biosciences |
| TUJ1 (βIII-tubulin) | 1:1000 | T8535 | mIgG2b | SIGMA |
| CLEAVED-CASPASE-3 | 1:1000 | 9664S | Rabbit IgG | Cell Signaling |
| PRO/CLEAVED-CASPASE-2 | 1:1000 | 2224S | mIgG1 | Cell Signaling |
| CLEAVED-CASPASE-8 | 1:1000 | 9496S | Rabbit IgG | Cell Signaling |
| BCL-2 | 1:1000 | 2876S | Rabbit IgG | Cell Signaling |
| BAX | 1:1000 | 2772S | Rabbit IgG | Cell Signaling |
| AIF | 1:1000 | 4642S | Rabbit IgG | Cell Signaling |
| GAPDH | 1:1000 | ab9484 | mIgG2b, κ | Abcam |
| FAS LIGAND | 1:1000 | 4273S | Rabbit IgG | Cell Signaling |
| FAS LIGAND | 1:100 | AF126 | Goat IgG | R & D systems |
| FAS NEUTRALIZING (FAS NT) | 300 ng/ml | 05-338 | mIgG1 | Millipore |
