# Supplementary material for: Inhibition of Apoptosis Blocks Human Motor Neuron Cell Death in a Stem Cell Model of Spinal Muscular Atrophy
Source: PLoS One. 2012 Jun 19;7(6):e39113. doi: 10.1371/journal.pone.0039113 (PMC3378532; doi:10.1371/journal.pone.0039113)
Supplement: Text S1 — Supporting materials and methods. (DOC) [file pone.0039113.s008.doc]

**Supporting Information (SI) Text**

**Inhibition of apoptosis blocks human motor neuron cell death in a stem cell model of spinal muscular atrophy**

### Dhruv Sareen, Allison D. Ebert, Brittany M. Heins, Jered V. McGivern, Loren Ornelas, Clive N. Svendsen

**SI Materials and Methods**

**Generation of SMA iPSCs using Episomal Plasmids**

Amaxa Human Dermal Fibroblast Nucleofector Kit was utilized to make the virus-free iPSC SMA lines. Briefly, GM09677 fibroblasts (0.8 x 10^6^ cells per nucleofection) were harvested and centrifuged at 200*g* for 5 minutes. The cell pellet was re-suspended carefully in Nucleofector Solution (VPD-1001, Lonza) and combined with pEP4-E02S-CK2M-EN2L and pEP4-E02S-ET2K vectors (Addgene) [1]. The cell/DNA suspension was transferred into the Nucleofector® and the U-022 program applied. The sample was gently transferred on to pre-warmed MEF feeder plates with the media or BD Matrigel™ Matrix coated 100 mm-dishes. The media was kept on for 48h and gradually changed to hiPSC media containing small molecules to enhance reprogramming efficiency. The small molecules used were, 1) selective inhibitor of G9a histone-methyl transferase (BIX01294, 1 µM), 2) glycogen synthase kinase 3β inhibitor of the Wnt/β-catenin signaling pathway (CHIR99021, 3 µM), 3) MEK pathway inhibitor (PD 0325901, 0.5 µM), 4) Selective inhibitor of TGF-β type I receptor ALK5 kinase, type I activin/nodal receptor ALK4 and type I nodal receptor ALK7 (A 83-01, 0.5 µM). All cultures were maintained under norm-oxygen conditions (5% O_2_). Colonies with ES/iPSC-like morphology appeared 25-31 days later. Subsequently, clones with the best morphology were picked, expanded and cryopreserved according to previously published protocols [2].

**iPSC characterization**

Quantitative RT-PCR for expression of pluripotency genes and conventional PCR for SMN expression were performed using primers and conditions as previously described [2, 3] (**Table S1**).Teratoma formation analysis was performed 6 weeks after an injection of ~ 2 million undifferentiated cells into SCID mice

**Neural Stem Cell Culture**

Briefly, iPSC colonies were gently lifted from MEF feeders by collagenase treatment for 20 min at 37^o^C and expanded in suspension cultures in poly-HEMA coated ultra-low attachment flasks with Stemline Neural Expansion Media (Sigma) supplemented with, EGF (100 ng/ml; Milipore), fibroblast growth factor-2 (FGF-2; 100 ng/ml; Milipore), and heparin (5 µg/ml; Sigma). NSC spheres rapidly formed and were passaged every 7-10 days by sectioning them into 200 µm chunks using an automated tissue chopper, as described previously [4]. At 3 weeks after the first passage following NSC spheres generation, the cultures reach a stable phase of growth where they predominantly contain Pax6 and Nestin positive NSCs. At this state, NSC spheres can be utilized for further differentiation protocols. All cultures were maintained at in humidified incubators at 37^o^C (5% CO_2_ in air).

**Motor Neuron Differentiation**

To induce motor neuron differentiation, NSC^EFH^ spheres were placed in neural induction medium (1:1 DMEM/F12 and 1% N2) in the presence of all-trans retinoic acid (RA; 0.1 µM ) for 1 week followed by the addition of purmorphamine (PMN; 1 µM) or Sonic Hedgehog (SHh; 10ng/ml) for another 1-3 weeks. Partially or fully dissociated spheres were then plated on poly-ornithine/laminin-coated coverslips in RA and PMN medium supplemented with 2% B27, db-cAMP, ascorbic acid (AA; 200 ng/ml), brain-derived neurotrophic factor (BDNF; 10 ng/ml) and glial cell line-derived neurotrophic factor (GDNF; 10 ng/ml) for a further 2–6 weeks. These conditions allowed for motor neuron differentiation under serum-free conditions. All differentiating cultures were maintained in humidified incubators at 37°C (5% CO_2_ in air). The culture medium was replenished every 3 days.

**Immunocytochemistry**

At the appropriate time point of differentiation in culture, plated cells were fixed in paraformaldehyde (PFA, 4% vol/vol) or chilled acetone:methanol (1:1), and rinsed in phosphate-buffered saline (PBS). Fixed cultures were blocked in 5-10% (vol/vol) goat or donkey serum with 0.2% (vol/vol) Triton X-100 and incubated with primary antibodies (**Table S2**). After incubation with the primary antibodies, cultures were rinsed in PBS and incubated in species specific AF488 or AF594-conjugated secondary antibodies. Nuclei were counterstained with Hoechst 33258 (0.5 µg/ml; Sigma) and mounted on glass slides using GelTol Aqueous Mounting Medium (Immunotech). Visualization was performed at 10X, 20X, 63X magnification (Nikon/Lecia) and quantification of positive cells was completed using Metamorph Offline software (Molecular Devices).

**Immunoblotting**

Cultures were washed twice with PBS and lysed in ice-cold lysis buffer [50 mM Tris-HCl (pH 7.5), 150 mM NaCl, 0.5% Nonidet P-40, 1 mM PMSF, 1 mM NaF, 1 mM DTT and 4 mg/ml complete protease inhibitor cocktail]. Protein concentration was determined in cell lysates using the Bio-Rad protein assay kit, separated with SDS-PAGE, transferred onto PVDF membrane and western blot analysis performed using standard protocols. Briefly, 10 µg protein extracts were denatured in Laemmli sample buffer followed by 5 minutes of boiling and then resolved on a 10% or 8% Tris-glycine gel (Novex, San Diego, CA). After electrophoresis (120 V for 2 hours), the proteins were transferred in 1x transfer buffer (25 mM Tris, 192 mM glycine, 0.1% SDS, and 20% methanol [
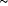
pH 8.4]) to a nitrocellulose membrane (Hybond-ECL; GE Healthcare; Piscataway, NJ), with constant current of 100 mA for 2 or 3 hours. The membranes were then blocked in 5% nonfat dry milk TBS solution for 1 hour at room temperature. The blots were incubated overnight at 4°C with the primary antibodies (**Table S2**). The membranes were washed three times with TBS solution including Tween-20 (TBS-T) incubated with horseradish peroxidase–linked donkey anti-mouse, donkey anti-goat or donkey anti-rabbit antibodies (Promega) for 2 hours at room temperature and then washed four times in TBS-T. Detection of the immunoreactive bands was performed with the ECL chemiluminescence detection kit (GE Healthcare).

**Determination of Apoptotic Cell Morphology**

Cells were seeded at a density of 50,000 cells/well on poly-ornithine and laminin coated coverslips, fixed with 4% paraformaldehyde for 45 minutes, washed with PBS, stained with 1 µg/mL Hoechst 33528 dye) (Invitrogen Probes, Eugene, OR) in PBS for 2 minutes at 37°C, and viewed under a fluorescence microscope (Leica). A nucleus with condensed chromatin and a discontinuous nuclear envelope was counted as an apoptotic cell. The percentage of apoptotic cells was calculated by counting the number of apoptotic cells in a viewing field relative to the total number of cells. Cell counts were determined over five viewing fields and averaged.

References

1. Yu J, Hu K, Smuga-Otto K, Tian S, Stewart R et al. (2009) Human induced pluripotent stem cells free of vector and transgene sequences. Science 324**:**797-801.

2. Yu J, Vodyanik MA, Smuga-Otto K, Antosiewicz-Bourget J, Frane JL et al. (2007) Induced pluripotent stem cell lines derived from human somatic cells. Science 318**:**1917-1920.

3. Ebert AD, Yu J, Rose FF, Jr., Mattis VB, Lorson CL et al. (2009) Induced pluripotent stem cells from a spinal muscular atrophy patient. Nature 457**:**277-280.

4. Svendsen CN, ter Borg MG, Armstrong RJ, Rosser AE, Chandran S et al. (1998) A new method for the rapid and long term growth of human neural precursor cells. J Neurosci Methods 85**:**141-152.
